# The complete chloroplast genome sequences of little millet (*Panicum sumatrense* Roth ex Roem. and Schult.) (Poaceae)

**DOI:** 10.1080/23802359.2018.1483771

**Published:** 2018-06-26

**Authors:** Raveendar Sebastin, Gi-An Lee, Kyung Jun Lee, Myoung-Jae Shin, Gyu-Taek Cho, Jung-Ro Lee, Kyung-Ho Ma, Jong-Wook Chung

**Affiliations:** a National Agrobiodiversity Center, National Institute of Agricultural Sciences, Jeonju-Si, Republic of Korea;; b Department of Industrial Plant Science and Technology, Chungbuk National University, Cheongju, Republic of Korea

**Keywords:** Chloroplast, little millet, Illumina sequencing, *P. sumatrense*

## Abstract

Little millet, *Panicum sumatrense* Roth ex Roem. & Schult., is an important cultivated species under the tribe Paniceae, sub-family Panicoideae and family Poaceae. In this study, for the first time we sequenced the complete chloroplast (cp) genome of *P. sumatrense* to investigate their phylogenetic relationship in the family Poaceae. The complete cp genome sequence of *P. sumatrense* is 139,384 bp in length with 38.6% overall GC content and exhibits a typical quadripartite structure comprising one pair of inverted repeats (22,723 bp) separated by a small single-copy region (12,583 bp) and a large single-copy region (81,355 bp). The *P. sumatrense* cp genome encodes 125 unique genes, which include 91 protein-coding genes, 4 rRNA genes, 30 tRNA genes, and 20 genes were duplicated in the inverted repeat region. This newly determined cp genome (*P. sumatrense*) could be valuable information for the breeding programs of this cereal crops in the family Poaceae.

## Introduction

The grass genus *Panicum* L., comprises of about 500 species distributed worldwide in tropical and subtropical regions, is one of the largest genera of the family Poaceae (Aliscioni et al. 2003). The great significance of this genus is almost certainly polyphyletic (Zuloaga 1987) in which the species with C_3_ and C_4_ photosynthetic systems (Brown 1977), and C_3_/C_4_ intermediate pathways (Zuloaga et al. 1998) have been reported. Though many dominating grasses found in the genus *Panicum*, common millet (*Panicum miliaceum*) and little millet (*Panicum sumatrense*) are economically important species within the genus (Baltensperger 1996). Understanding the relationships between the grasses is vital for germplasm conservation and their utilization. The cp genomes have been used in studies on plant phylogeograhy, genetic diversity, and evolution (Liu et al. 2016; Park 2016, 2017; Tsuruta et al. 2017). The cp genome of common millet was reported (Cao et al. 2017) and many other economically important *Panicum* cp genome were also available on pubic database. In this study, we report the chloroplast genome of little millet to determine its phylogenetic relationships within the family Poaceae.

The little millet seeds (Accession No. IT261894) were obtained from the Genebank division of National Institute of Agricultural Sciences, Republic of Korea. Seeds were germinated and fresh leaves were collected from 40-day-old seedlings. Total genomic DNA was extracted to build up genomic library and sequenced with pair-end (2 × 300 bp) by MiSeq instrument at LabGenomics (http://www.Lab.genomics.com/kor/). A total of 4,915,776 cleaned reads were obtained after quality trimming of raw reads and mapped with the reference cp genome, *A. sativa* L. (GenBank accession KM974733). The reference mapping produced 206,243 aligned reads with about an average 325× coverage. The complete circular cp genome was obtained from contig alignment and scaffolding of mapped reads. DOGMA (http://dogma.ccbb.utexas.edu/) software was used for annotation of protein-coding genes in the cp genome and manually inspected to predict transfer RNA (tRNA) and ribosomal RNA (rRNA) genes.

We determined the cp genome of *P. sumatrense* was 139,384 bp in length (NCBI accession number KX756177). The GC content was 38.6% which is similar to values previously reported with *Panicum* cp genomes. The LSC and SSC regions contained 81,355 bp and 12,583 bp, respectively, whereas the IR was 22,723 bp in length. The cp genome contained 125 known genes, including 91 protein-coding genes, 30 tRNA genes, and four rRNA genes. There were eight protein-coding genes, eight tRNA, and all four rRNA genes duplicated in the IR regions. Sixteen genes contained one or two introns, including the protein-coding genes, ycf3, petB, petD, atpF, ndhA, ndhB, rpl2, rpl16, rps12, and rps16. In addition, rps12 was identified as a trans-spliced gene.

**Figure 1.**
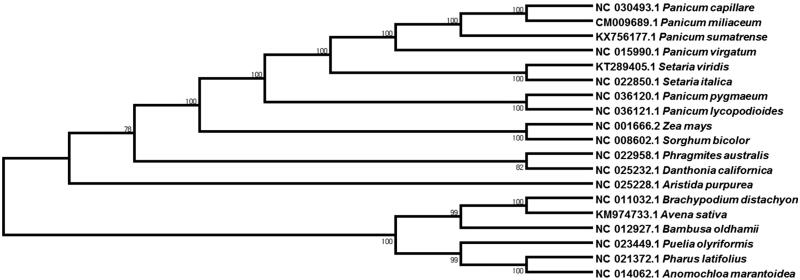
Phylogenetic relationships among 19 whole chloroplast genomes of Poaceae species. The complete chloroplast genome is downloaded from NCBI database and the phylogenetic tree is constructed by MEGA6 software.

The phylogenetic relationship of *P. sumatrense* with other species was evaluated with 19 Poaceae species by comparing the chloroplast genome. MAFFT v7.304 (Katoh and Standley 2016) program was used for the whole genome sequence alignment and MEGA6 (Tamura et al. 2013) software was used to construct a maximum likelihood (ML) tree with 1000 bootstrap replicates. The phylogenetic tree showed that the little millet, *P. sumatrense* was clustered with the most closely related common millet, *P. miliaceum* and witchgrass, *Panicum capillare* (Figure 1). The newly determined cp genome will provide essential data for further study on the phylogeny and evolution of the genus *Panicum* in the family Poaceae.

## References

[CIT0001] Aliscioni SS, Giussani LM, Zuloaga FO, Kellogg EA 2003 A molecular phylogeny of *Panicum* (Poaceae: Paniceae): tests of monophyly and phylogenetic placement within the Panicoideae. Am J Bot. 90:796–821. 21659176 10.3732/ajb.90.5.796

[CIT0002] Baltensperger DD 1996 Foxtail and proso millet In: Janick J, editor. Progress in new crops. Alexandria, VA: ASHS Press; p. 182–190.

[CIT0003] Brown WV 1977 The Kranz syndrome and its subtypes in grass systematics. Mem Torrey Bot Club. 23:1–97.

[CIT0004] Cao XN, Wang JJ, Wang HG, Liu SC, Chen L, Tian X, Qin HB, Wang L, Na XF, Qiao ZJ 2017 The complete chloroplast genome of *Panicum miliaceum*. Mitochondrial DNA B. 2:43–45. English. 10.1080/23802359.2016.1157773 PMC7799612 33473711

[CIT0005] Katoh K, Standley DM 2016 A simple method to control over-alignment in the MAFFT multiple sequence alignment program. Bioinformatics. 32:1933–1942. 27153688 10.1093/bioinformatics/btw108 PMC4920119

[CIT0006] Liu F, Tembrock LR, Sun C, Han G, Guo C, Wu Z 2016 The complete plastid genome of the wild rice species *Oryza brachyantha* (Poaceae). Mitochondrial DNA B. 1:218–219. 10.1080/23802359.2016.1155093 PMC7871827 33644346

[CIT0007] Park T-H 2016 The complete chloroplast genome sequence of potato wild relative species, *Solanum nigrum*. Mitochondrial DNA B. 1:858–859. 10.1080/23802359.2016.1250133 PMC7800014 33473656

[CIT0008] Park T-H 2017 The complete chloroplast genome of *Solanum berthaultii*, one of the potato wild relative species. Mitochondrial DNA B. 2:88–89. 10.1080/23802359.2017.1285213 PMC7800855 33473725

[CIT0009] Tamura K, Stecher G, Peterson D, Filipski A, Kumar S 2013 MEGA6: molecular evolutionary genetics analysis version 6.0. Mol Biol Evol. 30:2725–2729. 24132122 10.1093/molbev/mst197 PMC3840312

[CIT0010] Tsuruta S-I, Ebina M, Kobayashi M, Takahashi W 2017 Complete chloroplast genomes of *Erianthus arundinaceus* and *Miscanthus sinensis*: comparative genomics and evolution of the saccharum complex. Plos One. 12:e0169992. 28125648 10.1371/journal.pone.0169992 PMC5268433

[CIT0011] Zuloaga FO, 1987 Systematics of new world species of Panicum (Poaceae) In: Soderstrom TR, et al. editors. Grass systematics and evolution. Wash. DC: Smithsonian Institution Press; p. 287–306.

[CIT0012] Zuloaga FO, Morrone O, Vega AS, Giussani LM 1998 Revision y analysis cladistico de steinchisma (Poaceae: Panicoideae: Paniceae). Ann Missouri Bot Gard. 85:631–656.

